# Got Milk? How Freedoms Evolved From Dairying Climates

**DOI:** 10.1177/0022022118778336

**Published:** 2018-06-11

**Authors:** Evert Van de Vliert, Christian Welzel, Andrey Shcherbak, Ronald Fischer, Amy C. Alexander

**Affiliations:** 1 University of Groningen, The Netherlands; 2 Leuphana University of Lüneburg, Germany; 3 National Research University–Higher School of Economics, St. Petersburg, Russia; 4 Victoria University of Wellington, New Zealand; 5 University of Gothenburg, Sweden

**Keywords:** lactose tolerance, gene-culture coevolution, climato-economic, encultured freedoms, thermo-hydraulic theory

## Abstract

The roots and routes of cultural evolution are still a mystery. Here, we aim to lift a corner of that veil by illuminating the deep origins of encultured freedoms, which evolved through centuries-long processes of learning to pursue and transmit values and practices oriented toward autonomous individual choice. Analyzing a multitude of data sources, we unravel for 108 Old World countries a sequence of cultural evolution reaching from (a) ancient climates suitable for dairy farming to (b) lactose tolerance at the eve of the colonial era to (c) resources that empowered people in the early industrial era to (d) encultured freedoms today. Historically, lactose tolerance peaks under two contrasting conditions: cold winters and cool summers with steady rain versus hot summers and warm winters with extensive dry periods (Study 1). However, only the cold/wet variant of these two conditions links lactose tolerance at the eve of the colonial era to empowering resources in early industrial times, and to encultured freedoms today (Study 2). We interpret these findings as a form of gene-culture coevolution within a novel thermo-hydraulic theory of freedoms.

## Introduction

Why are inhabitants of some nations freer than others? An easy answer is that the countries’ elites make all the difference, and that the masses just follow the overarching choice whether or not to institutionalize freedoms. A more serious, Nobel-Prize-winning answer (Sen, 1999), adopted by the United Nations (see for example, United Nations Development Programme [UNDP], 2004), is that economic prosperity provides freedoms. A third view has it that “good institutions” cause both economic prosperity and cultural freedoms (Acemoglu & Robinson, 2012; Fukuyama, 2012; North, Wallis, & Weingast, 2009).

A joint problem of all three of these approaches is that they might falsely attribute habits of freedom to the characteristics of inhabitants rather than their habitats (Jones & Nisbett, 1971). Accordingly, these explanations neglect the possibility that thermal features of climate (Acemoglu, Johnson, & Robinson, 2001; Van de Vliert, 2013a), as well as precipitational features of climate (Welzel, 2013, 2014), have remotely shaped the more proximate impact of both wealth and institutions on contemporary freedoms. A look at climatically selective settlement patterns during colonial history illustrates why this is likely to be a crucial omission.

Indeed, the role of climate is apparent in the colonial settlement strategies of European emigrants over the last five centuries. Western Europeans preferred to migrate from their home country to overseas areas with a similar climate because this meant that they encountered familiar hardships and resources and could pursue a similar mode of how to make a living (e.g., North America, South Africa, Australia, and New Zealand). This also meant widespread settlement of these emigrants, which came at the cost of mass displacement and marginalization of the native population (although not needed from a colonial perspective). On the flip side, Western Europeans settled in much smaller numbers in tropical areas with unfamiliar existential threats and challenges.

Climatic circumstances not only shaped settlement patterns but also shaped institutional legacies after settlement had happened (Acemoglu et al., 2001; Acemoglu & Robinson, 2012; Engerman, 2007; Sokoloff & Engerman, 2000; Welzel, 2013). In the Europe-like climate zones characterized by cold winters, steady rain, and high seasonality, European emigrants could easily marginalize native populations and then replicate and advance their home-style liberating institutions, designed to grant rights and freedoms to the settlers. In sharp contrast, in climatically demanding and often life-threatening zones characterized by hot summers, unsteady rain, and low seasonality, the relatively small number of privileged European colonizers set up *coercive* institutions, designed to enslave the native populations and to efficiently extract resources.

A similarly intriguing case of selective migration unfolded around 6,500 years ago, when climatic conditions allowed Neolithic peoples to start to establish well-developed dairy economies in western and northern Europe. Curry (2013), who calls it the milk revolution, concludes in *Nature*: “When a single genetic mutation first let ancient Europeans drink milk, it set the stage for a continental upheaval” (p. 20), the remnants of which are thought to still be visible today. In essence, Curry postulates that the unique combination of dairying climates and the ability to drink milk throughout the adult life is a long-term precursor of economic development and human empowerment. This is, however, a far-fetched idea in need of closer scrutiny as climates and genes only set boundary conditions for societal development, rather than completely determining developmental trajectories.

The present empirical exploration is not limited to Europe. It is limited, though, to Old World civilizations in Africa, Asia, and Europe because societal development in the Americas and Oceania unfolded on a playing field that is categorically different from the Old World. The reason is that colonialization and mass immigration by Europeans has fundamentally revamped the demographic setup of the New World, with large-scale replacement and marginalization of the local populations and the establishment of derivatives of European settlement patterns and institutions that protected the rights and freedoms of the settlers (Acemoglu et al., 2001; Diamond, 1997; Sokoloff & Engerman, 2000). Hence, population history places the New World out of comparison with the dynamics differentiating the civilizations of the Old World.

We begin with a systematic exploration of the millennia-long coevolution of dairying cultures and lactose tolerance in particular local climates around the globe suitable for dairy farming. Building on the results, we continue with a historical process analysis of lactose tolerance at the eve of the colonial era (i.e., around 1500 CE), empowering resources in early industrial times (i.e., around 1800), and encultured freedoms in the information age of today (i.e., around 2000). Both parts of this conceptual and empirical research trajectory, conveniently indicated as Study 1 and Study 2, are in essence analyses of gene-culture coevolution and niche construction (Durham, 1991; Laland, Odling-Smee, & Myles, 2010; O’Brien & Laland, 2012; Richerson & Boyd, 2008).

## Lactose Tolerance in Dairying Climates (Study 1)

Human populations worldwide differ in the prevalence of genotypic lactase persistence (Cook, 2014; Gerbault, Moret, Currat, & Sanchez-Mazas, 2009; Ingram, Mulcare, Itan, Thomas, & Swallow, 2009), phenotypic lactose tolerance (Cook, 2014; Durham, 1991), and habituated milk consumption (Curry, 2013; FAOSTAT, 2014). People’s capacity to absorb milk is more widespread under three conditions: (a) at higher latitudes, where insufficient ultraviolet-B radiation causes deficiencies of vitamin D_3_ and calcium (Durham, 1991; Gerbault et al., 2009; Itan, Powell, Beaumont, Burger, & Thomas, 2009); (b) in arid and torrid areas where fresh water scarcity turns milk into a welcome source of hydration (Cook & Al-Torki, 1975; Ingram et al., 2009); and (c) in pastoral environments where cattle herding provides abundant milk supplies (Bloom & Sherman, 2005; Durham, 1991; Gerbault et al., 2009; Ingram et al., 2009). We argue, and then show, that these three occurrences of lactose tolerance can be integrated into a single model of culturally evolved dairying climates.

### A Thermo-Hydraulic Theory

Our explanatory model builds on (a) the axiom that cold winters and hot summers cause thermal stress in plants, in milk-producing cattle feeding on plants, and in humans feeding on both plants and animals, (b) the corollary that 22°C (~72°F) is an appropriate reference point against which to measure cold stress and heat stress (Cline, 2007; Hatfield & Prueger, 2015; Van de Vliert, 2017), and (c) the fact that the planetary system has, for millennia, offered more cold stress than heat stress (Ditlevsen, Svensmark, & Johnsen, 1996; Van de Vliert & Tol, 2014), thus more steady rain (Oliver, 1996), relegating hydraulic stress to climates with heat stress. Across the 108 countries in the current study, minimal monthly precipitation (source: Parker, 1997) is lower in countries with heat stress (source: Van de Vliert, 2013b; main effects: *B* = −.14, *p* = .32, for cold stress; *B* = −.58, *p* < .001 for heat stress), and lowest in countries with both cold stress and heat stress (interaction effect: *B* = −.36, *p* < .01; Δ*R*^2^= .05, total Δ*R*^2^= .24).

Small deviations from 22°C, typical for climates with mild winters and summers and steady rain, allow for a wide variety of subsistence modes, including horticulture, multi-crop agriculture, and dairying. Consequently, the evolutionary benefits of cattle husbandry, milk consumption, and lactose tolerance are modest at best (Bloom & Sherman, 2005; Durham, 1991), especially where fishing serves as an extra source of subsistence (e.g., Micronesia, Seychelles). By contrast, large seasonal deviations from 22°C prevail in arctic climates with at least three cold seasons and much snow and ice, in desert climates with at least three hot seasons and hydraulic stress, and in continental climates with both cold winters and hot summers. All of these climates make it difficult to eke out a living from hunting and gathering, while farming options are inherently limited. Staying put in such extremely stressful climates requires adaptations other than developing lactose tolerance and consuming milk. The Inuit peoples in the arctic North illustrate the point. Instead of dairying economies and tolerance of lactose, they evolved fishing economies and tolerance of cod-liver oil (Bloom & Sherman, 2005; Durham, 1991).

The true evolutionary utility of lactose tolerance resides in medium seasonal deviations from the optimal livability zone around 22°C. Two contrasting climatic constellations fall into this rubric: cold winters and cool summers, causing steady rain, versus hot summers, and warm winters, causing hydraulic stress. Both constellations endanger the satisfaction of nutritional and health needs by a lack of crop yields during at least one season (Cline, 2007; Hatfield & Prueger, 2015). Livestock farming can solve this problem by offering dairy products and meat during the one fallow season. The low prevalence of cattle diseases during either extreme winters or extreme summers makes this option even more viable (Bloom & Sherman, 2005). Hence, regions with either cold/wet conditions or hot/dry conditions place an evolutionary premium on lactose tolerance and milk consumption.

### Methods and Measures

The earliest available data on lactose tolerance from around 1500 CE (source: Cook, 2014) allowed for a tentative test of the thermo-hydraulic hypothesis across 108 Old World countries, assuming three premises. First, the time around 1500 is of great historic significance as it is located at the eve of the colonial era when differences between the Old World’s agrarian civilizations began to turn into determinants of global power positioning (Acemoglu et al., 2001; Diamond, 1997). Second, the geographic distribution of the major clusters of world climates underwent negligible changes for millennia, even if one takes into account smaller climatic fluctuations such as the Medieval Warming Period and the Little Ice Age (Oliver, 1996). Third, and because of that, the near-stability of climate relations among spatial areas enables us to use 20th-century climate measures as crude proxies of climate relations *long before* the 15th century, providing a conservative test of the thermo-hydraulic theory.

Against these premises, we operationalize a country’s cold stress as the sum of the absolute downward deviations in centigrade from 22°C for the average lowest temperature in the coldest month, the average highest temperature in the coldest month, the average lowest temperature in the hottest month, and the average highest temperature in the hottest month. Likewise, a country’s heat stress is the sum of the absolute upward deviations in centigrade from 22°C for the same four average temperatures (source: Van de Vliert, 2013b). Steady rain is proxied by the minimal monthly precipitation divided by the maximal monthly precipitation (source: Parker, 1997) because annual precipitation has a more steady rhythm to the extent that the monthly minimum is higher and the monthly maximum is lower. It is important to realize that two large countries in our sample have multiple climatic subzones (China = seven, India = four; Cline, 2007), so that single scores for climatic predictors induce error leading to an underestimation of the “true” effects of climate. For each of the 108 Old World countries in the sample, Table S1 in the supplementary material provides the indices used for cold stress, heat stress, steady rain, and lactose tolerance in 1500 (for intercorrelations, see Table 1).

**Table 1.** Correlations Among Main Variables (108 Countries).

Main variable	1	2	3	4	5
1. Cold stress					
2. Heat stress	−.68***				
3. Steady rain	.67***	−.60***			
4. Lactose tolerance in 1500	.41***	−.33***	.65***		
5. Empowering resources in 1800	.51***	−.46***	.70***	.62***	
6. Encultured freedoms in 2000	.42***	−.46***	.62***	.66***	.70***

*** *p* < .001 (two-sided tests).

**Figure 1.**
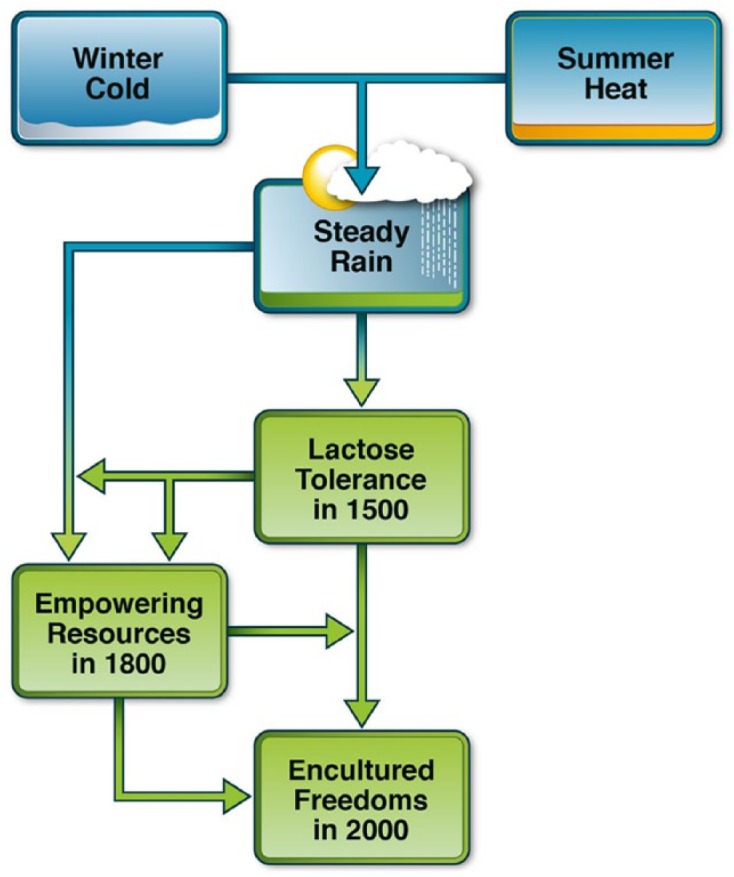
The thermo-hydraulic theory of freedoms. *Note.* The theory proposes that (a) thermo-hydraulic stress has historically shaped lactose tolerance in 1500 which, over subsequent centuries, (b) has first interacted with steady rain in shaping empowering resources in 1800, and (c) has then interacted with empowering resources in shaping encultured freedoms in 2000.

Next, we perform country-level regression analyses, using cold and heat stress as ultimate predictors, steady rain as a mediator, and prevalence levels of lactose tolerance in 1500 as the dependent variable (visualization in the upper part of Figure 1). More specifically, employing 5,000 bootstrap samples for constructing bias-corrected confidence intervals, and applying Hayes’s (2013) Diagram 8 to standardized country-level variables, we estimate the direct effects and the rain-mediated effects of cold stress, heat stress, and their interaction on lactose tolerance in 1500.

### Results and Discussion

**Figure 2.**
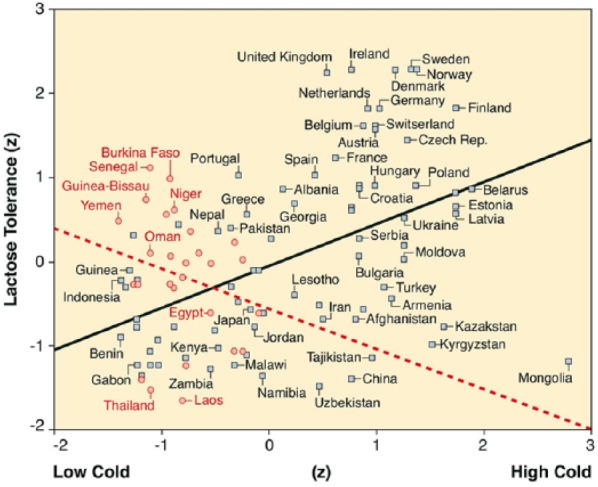
Interactive effects of cold stress and heat stress on lactose tolerance in 1500. *Note.* Represented are rain-mediated effects (*R*^2^ = .465) of higher cold stress and low heat stress (squares and solid upward slope for 81 Old World countries), supplemented with direct effects of higher cold stress and high heat stress (circles and broken downward slope for 27 Old World countries; the 81 to 27 split is chosen because other splits provide less telling illustrations of the regression equation)^1^.

Across 108 Old World countries, cold stress and heat stress have an interaction effect on lactose tolerance in 1500 (Table 2, Model 1) that is mediated by steady rain (Model 2 including Footnote d) and robust against adjustments for geographic and cultural proximity of countries within climatic regions (Model 3). A visual inspection of the interaction effect reveals that lactose tolerance indeed peaks under the contrasting conditions of one harsh season with either cold/wet weather (Figure 2, upper right; for example, Ireland, Denmark, and Sweden) or hot/dry weather (Figure 2, upper left; for example, Senegal, Guinea-Bissau, and Yemen), and is much lower under other climatic conditions (for example, Gabon, Uzbekistan, and Mongolia in the lower part of Figure 2).

**Table 2.** Rain-Mediated Effects of Cold Stress (CS) and Heat Stress (HS) on Lactose Tolerance in 1500 (108 Countries).

Regression model	Model 1	Model 2	Model 3	Model 4	Model 5
Predictor^a^	*B* ^b^	*B*	*B* ^c^	*B*	*B*
Cold stress (CS)	.051	−.149	−.132	−.299	−.672***
Heat stress (HS)	−.369**	−.089	−.008	−.064	−.219
CS × HS	−.469***	−.275*	−.164*	−.299*	−.298**
Steady rain^d^		.647***	.658***	.538***	−.226**
Arable land^e^				.119	
Nonzoonotic diseases^f^				−.217	
Zoonotic diseases				−.193	
Latitude (LAT)^g^					.862***
Longitude (LON)^h^					−.495***
LAT × LON					−.361*
Δ*R*^2^	.266***	.036		.079**	.131***
Total *R*^2^	.266***	.465***	.414***	.544***	.675***

a Predictors are standardized variables and products of standardized variables.

b *B*s shown are unstandardized regression coefficients. There is no problematic multicollinearity, neither in Models 1 to 3 (Variance inflation factors ≤ 3.073), nor in Models 4 and 5 (Variance inflation factors ≤ 8.841), and there are no outliers (Cook’s distances ≤ .164).

c In a conservative reanalysis, we checked whether the interaction effect of thermo-hydraulic stress on lactose tolerance in 1500 results from a violation of the assumption of independent observations because 26 small and geographically adjacent countries, nested in seven climatic regions (source: Cline, 2007), have similar thermo-hydraulic circumstances and subsistence conditions. Regression weights were assigned on the basis of the number of countries in each region. Specifically, we assigned a weight of .143 to seven countries in Southeastern Europe (Albania, Bosnia and Herzegovina, Bulgaria, Croatia, Macedonia, Serbia, and Slovenia), a weight of .250 to four countries in Scandinavia (Denmark, Finland, Norway, and Sweden), a weight of .250 to four countries in Central Europe (Austria, Czech Republic, Hungaria, and Switzerland), a weight .333 to three countries in Central Asia (Kyrgyzstan, Tajikistan, and Turkmenistan), a weight of .333 to three countries in West Africa (Guinea, Guinea-Bissau, and Liberia), a weight of .500 to two countries in Equatorial Africa (the Congo’s and Gabon), a weight of .500 to two countries in Southern Africa (Botswana and Namibia), and a weight of 1.000 to countries that are not problematically nested in a climatic region.

d Cold deviations from 22°C (*B* = .308, *p* = .028), heat deviations from 22°C (*B* = −.434, *p* < .001), and their interaction (*B* = −.300, *p* = .009) are determinants of steady rain (*R*^2^ = .526). In addition, as reflected in Models 1 to 3, steady rain mediates the relationship between thermal stress and lactose tolerance (mediation effect = −.194; confidence intervals: lower limit = −.382, upper limit = −.066).

e The percentage of available arable land is retrieved from Parker (1997).

f The prevalence of nonzoonotic and zoonotic parasitic diseases is retrieved from Fincher and Thornhill (2012).

g Midrange distance (in latitude degrees) from the geographic equator represents a set of rival predictors including geomagnetic field, daylength variation, and parasitic disease burden.

h Midrange distance (in longitude degrees) from the Greenwich meridian (west is negative, east is positive) represents another set of rival predictors including electronic density, state antiquity, religion, and fertility pattern.

* *p* < .05. **p < .01. ***p < .001 (two-sided tests).

The solid upward slope in Figure 2 indicates that lactose tolerance is more widespread in countries with colder winters and cool summers. The effect is indirect because steady rain mediates the impact of temperature on lactose tolerance (Lower Limit Confidence Interval [LLCI] = .25, Upper Limit Confidence Interval [ULCI] = .61; direct: LLCI = −.19, ULCI = .44). By contrast, the downward slope for decreasing lactose tolerance in countries with colder winters and hot summers represents a direct effect (LLCI = −.83, ULCI = −.02), not an indirect effect through steady rain (LLCI = −.26, ULCI = .24). In other words, rain is an important mediating factor for explaining lactose tolerance, but only in cold climates. These climatic effects cannot be explained away by arable-land availability or parasitic diseases (Table 2, Model 4), nor by numerous variables related to latitude and longitude, including North-South and East-West variations in lactose tolerance (Model 5).

The *calcium absorption hypothesis* stating that deficient ultraviolet-B radiation at colder latitudes causes more lactose tolerance (Durham, 1991; Gerbault et al., 2009; Itan et al., 2009) does not explain the decreasing lactose tolerance in countries with colder winters and hot summers (downward slope in Figure 2). The *arid environment hypothesis* stating that inhabitants of steppes and deserts with limited water resources are bound to drink milk (Cook & Al-Torki, 1975; Ingram et al., 2009) is at odds with the decreasing lactose tolerance in drier countries with cold winters and hotter summers (LLCI = −.80, ULCI = −.22 for the vertical slope difference at the right of Figure 2). The *pastoralist hypothesis* stating that steppe populations have adapted to cattle milk (Bloom & Sherman, 2005; Durham, 1991; Gerbault et al., 2009; Ingram et al., 2009) fails to explain why lactose tolerance is low in pasture-friendly countries with mild winters and summers and not too unsteady rain (e.g., Kenya and Liberia). Thus, our thermo-hydraulic model is the first one to plausibly unify the scattered evidence for the three leading explanations of lactose tolerance.

In validation of these findings, a further examination shows that cold/wet conditions explain why dairying practices and lactose tolerance spread from the Middle East to Europe, and not to East Asia, as part of the Neolithic transition from hunting and gathering to agriculture (Curry, 2013). Indeed, the 32 European countries in Table S1 have climates with more cold stress (*M*_cold stress_ = 66) than the 21 East-Asian countries (*M*_cold stress_ = 37; *t* = 4.46, *p* < .001). On top of the colder conditions, the European countries also have steady rain (*M*_monthly minimum_ = 33 mm, *M*_monthly maximum_ = 88 mm), whereas the East-Asian countries have more alternation of drought (*M*_monthly minimum_ = 18 mm; *t* = 2.78, *p* < .01) and deluge (*M*_monthly maximum_ = 258 mm; *t* = −6.15, *p* < .001). These differences suggest that Europeans have evolved more lactose tolerance indeed for the reasons stated by the thermo-hydraulic theory.

## Lactose Tolerance, Resources, and Freedoms (Study 2)

The results of Study 1 support the idea that past climatic conditions contributed to both culturally transmitted dairy farming and genetically transmitted lactose tolerance (Durham, 1991). It might be reasonable to expect that this manifestation of gene-culture coevolution will continue to influence societal development, kicking off a new cycle of human niche construction (cf. Laland et al., 2010; O’Brien & Laland, 2012; Richerson & Boyd, 2008), although we are left in the dark as to the actual course of such future events. Aiming to somewhat dispel that darkness, Study 2 explores and refines Curry’s (2013) speculation that the unique combination of European climate and lactose tolerance is a long-term precursor of societal development, most notably the European progression toward human empowerment.

Curry (2013) seems to deem it important that human babies create a steadily widening spectrum of freedoms of movement and development by consuming and digesting milk (see also Durham, 1991), and that one-third of grown-ups keep this process of freedom creation going in several European, West African, and South-West Asian pockets of lactose tolerance (see also Bloom & Sherman, 2005; Cook & Al-Torki, 1975; Durham, 1991; Itan et al., 2009). As visualized in the lower part of Figure 1, the thermo-hydraulic theory of freedoms specifically proposes that the cycle of cumulative niche construction consists of three entwined processes: a mediating process, a matching process, and a self-reinforcing process.

### Three Processes of Niche Construction

#### The mediating process

Steady rain is a vital resource as every society depends on water supplies for sustaining health (daily drink, food, and hygiene), and creating wealth (economic production). Given that steady rain also modulates lactose tolerance, it would not come as a surprise if lactose tolerance were to serve as an intermediary affecting health, wealth, and other societal offshoots. Lactose absorption is indeed conducive to better health and longer life expectancy (Birnie et al., 2012; Curry, 2013; Durham, 1991), which encourages investments in goals with a delay of gratification including wealth accumulation. The orientation of life strategies toward the long-term well-being of individuals is also clearly visible in time for personal development gained from postponed parenthood (Welzel, 2013, 2014).

As empowering resources, health, wealth, and postponed parenthood, in turn, are expected to provide both women and men with the capability to undertake activities of their choice, be it on their own or in unison with others (Acemoglu et al., 2001; Sen, 1999; Van de Vliert, 2013a; Welzel, 2013, 2014). The societal outcomes of centuries-long processes of learning to pursue goals and transmit values and practices based on autonomous individual and collective choices are defined here as encultured freedoms. Typically, the enculturation of freedoms also means that differences between groups such as men versus women and higher-ups versus lower-downs become increasingly less important in a society’s cultural expressions (Alexander & Welzel, 2011; Midlarsky & Midlarsky, 1997; Welzel, 2013, 2014).

#### The matching process

There has been much debate among statisticians about whether a variable *M* can simultaneously mediate and modify an effect of an independent variable *X* on a dependent variable *Y* (for an overview of the debate illustrated with examples, see Hayes, 2013). The upshot of this debate is that most scholars nowadays are comfortable with the idea that a variable *X* (e.g., ingroup love) can reinforce its own influence on *Y* (e.g., outgroup hate) by creating an amplifying intermediary condition (e.g., feelings of moral superiority; Brewer, 1999). In the matching part of the thermo-hydraulic theory of freedoms, the independent variable *X* is steady rain, the mediator and modifier *M* is lactose tolerance in 1500, and the dependent variable *Y* is empowering resources in 1800.

Simultaneously with the mediating process, the matching process is expected to fine-tune the influence of steady rain on the prevalence of lactose tolerance when paving the way to resources and freedoms. Viewed from their perspective, lactose-tolerant people try to acquire more resources and create greater freedoms if: (a) cold stress motivates them to undertake productive activity in providing heating, shelter, housing, clothing, and food storage for the nongrowing winter season (Parsons, 2003); (b) opportunities exist for generating agrarian surplus, especially so in regions with steady rain owing to cold winters and cool summers (Cline, 2007; Masters & Wiebe, 2000; Midlarsky & Midlarsky, 1997; Welzel, 2014); (c) they are not victimized by heat stress as a paralyzing burden that discourages productive activity (Parsons, 2003); and (d) they are not forced to cope with centralized elite control over water resources which is typical of agrarian economies in hot/dry regions but relatively absent in cold/wet regions (Masters & Wiebe, 2000; Midlarsky & Midlarsky, 1997; Welzel, 2014).

#### The self-reinforcing process

In the third and final part of the thermo-hydraulic theory of freedoms, lactose tolerance in 1500 (*X*) is expected to have strengthened its own effect on encultured freedoms in 2000 (*Y*) to the extent that lactose-tolerant generations of people have been empowering themselves with resources (*M* in 1800). This is a societal-level rather than individual-level process. Although growing toddlers in lactose-tolerant populations continue to consume milk and to thus enjoy a somewhat better health and somewhat greater opportunities to make free and autonomous choices, that is predicted to be a trivial small-scale development unless it is empowered later on by the societal resources of a longer life expectancy, a higher income, and postponed parenthood. Conversely, even adults who do not consume dairy products themselves but who are members of lactose-tolerant populations, may still benefit from the centuries-long accumulation of resources and opportunities.

The core idea is that the collective intergenerational transmission of lactase persistence in cold/wet and hot/dry climates (Figure 2) creates a phenotype that can facilitate freedom of choice and action under resourceful conditions. In elegant accord with cultural niche construction theory (Laland et al., 2010; O’Brien & Laland, 2012), the societal availability of beneficial resources—in cold/wet rather than hot/dry regions—is thought to complement the collective genetic ability to benefit from dairy consumption. Specifically, we hypothesize that the very modest direct effect of lactose tolerance on the enculturation of freedoms amplifies itself under the empowering conditions of health, wealth, and postponed parenthood.

### Methods and Measures

The entwined mediating, matching, and self-reinforcing processes are major components of the thermo-hydraulic theory of freedoms (Figure 1)—a longitudinal theory that requires testing salient time points in the history of civilizations. With a view to amending the incomplete insight that economic development drives freedom (Sen, 1999), we model temporality by a sequential ordering of major socio-economic eras around three time points—1500, 1800, and 2000. As already mentioned, the year 1500 at the eve of the colonial era is used for measuring lactose tolerance in Old World countries (Cook, 2014) with differing thermo-hydraulic climates (Ditlevsen et al., 1996; Oliver, 1996; Parker, 1997; Van de Vliert, 2013b).

The year 1800 is chosen as representing the beginning of the Industrial Revolution and of historically unprecedented differences among countries in standards of living and available resources (Acemoglu et al., 2001; Maddison, 2007; Welzel, 2013). Empowering resources in 1800 are approximated by the average of three standardized country measures: child survival (health), per capita income (wealth), and fewer children per family (postponed parenthood) (source: https://www.gapminder.org). Finally, the year 2000 is chosen to represent the socio-economic turning point toward the information age of today. The outcome variable, encultured freedoms in 2000, summarizes standardized measures of (a) emancipative values emphasizing free choice and equal opportunities (source: Welzel, 2013); (b) freedom from discrimination (source: Van de Vliert, 2011a); (c) press freedom (source: Van de Vliert, 2011b); and (d) political rights and civil liberties (source: Pemstein, Meserve, & Melton, 2010).

Detailed country scores for dairying climates and lactose tolerance in 1500 (Table S1), and for empowering resources in 1800 and encultured freedoms in 2000 (Table S2) are provided in the supplementary material (for intercorrelations, see Table 1). In a first country-level analysis, we regress empowering resources in 1800 on cold stress, heat stress, and their interaction (Table 3, Model 1), then subsequently add steady rain (Model 2), lactose tolerance in 1500 (Model 3), and the temporal interaction of steady rain and lactose tolerance (Model 4). In a similar second analysis, we regress encultured freedoms in 2000 on the climatic predictors (Table 4, Model 1), then subsequently add lactose tolerance in 1500 (Model 2), empowering resources in 1800 (Model 3), and the temporal interaction of lactose tolerance and empowering resources (Model 4).

**Table 3.** Effects of Steady Rain (SR) and Lactose Tolerance in 1500 (LT) on Empowering Resources in 1800, Taking Account of Thermal Stress (108 Countries).

Regression model	Model 1	Model 2	Model 3	Model 4	Model 5
Predictor^a^	*B* ^b^	*B*	*B*	*B*	*B* ^c^
Cold stress (CS)	.274*	.070	.116	.202	.175
Heat stress (HS)	−.295*	−.007	.021	.129	.158
CS × HS	−.136	.063	.149	.213*	.209
SR		.665***	.464***	.265*	.324*
LT			.311***	.291***	.235***
SR × LT				.320***	.320***
Δ*R*^2^	.289***	.210***	.052***	.073***	
Total *R*^2^	.289***	.499***	.551***	.624***	.564***

a Predictors are standardized variables and products of standardized variables.

b *B*s shown are unstandardized regression coefficients. There is no multicollinearity (Variance inflation factors ≤ 3.646), and there are no outliers (Cook’s distances ≤ .224).

c Conservative reanalysis (see note c in Table 2).

* *p* < .05. **p < .01. ***p < .001. (two-sided tests).

**Table 4.** Effects of Lactose Tolerance in 1500 (LT) and Empowering Resources in 1800 (ER) on Encultured Freedoms in 2000, Taking Account of Thermo-Hydraulic Stress (108 Countries).

Regression model	Model 1	Model 2	Model 3	Model 4	Model 5
Predictor^a^	*B* ^b^	*B*	*B*	*B*	*B* ^c^
Cold stress (CS)	−.192	−.133	−.179	−.139	−.142
Heat stress (HS)	−.313**	−.278**	−.286**	−.236*	−.222*
CS × HS	−.227*	−.117	−.176	−.169	−.131
Steady rain	.457***	.200	.019	−.015	.044
LT		.397***	.275***	.235***	.198*
ER			.481***	.310**	.310
LT × ER				.163**	.126*
Δ*R*^2^	.428***	.104***	.085***	.026**	
Total *R*^2^	.428***	.532***	.617***	.643***	.580***

a Predictors are standardized variables and products of standardized variables.

b *B*s shown are unstandardized regression coefficients. There is no multicollinearity (Variance inflation factors ≤ 3.609), and there are no outliers (Cook’s distances ≤ .153).

c Conservative reanalysis (see Note c in Table 2).

* *p* < .05. **p < .01. ***p < .001. (two-sided tests).

### Results and Discussion

**Figure 3.**
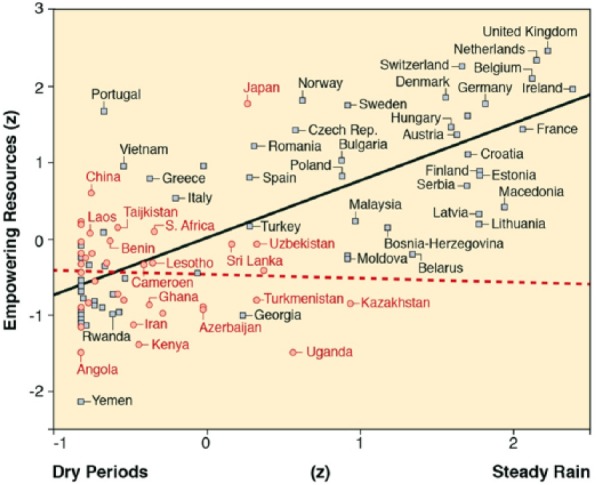
Interactive effects of steady rain and lactose tolerance in 1500 on empowering resources in 1800. *Note.* Represented are results while controlling for thermal stress (*R*^2^ = .624). Steady rain has no effect where lactose tolerance is low (circles and broken horizontal slope for 43 Old World countries; *r* = −.038, *p* = .809), but a strong effect where lactose tolerance is high (squares and solid upward slope for 65 Old World countries; *r* = .759, *p* < .001; the 43 to 65 split is chosen because other splits provide less telling illustrations of the regression equation)^1^.

At the end of the 18th century, empowering resources were more abundant in places where steadier rain makes water more universally and permanently available, and in places with historically higher levels of lactose tolerance (Table 3, Model 3). Beyond these additive effects, there is the important conditionality that steadier rain had a substantially stronger effect on the abundance of empowering resources at the end of the 18th century in places with high levels of lactose tolerance in 1500 (Table 3, Model 4; Figure 3). Conditional process analysis (Hayes, 2013, Diagram 74 with 5,000 bootstrap samples for constructing bias-corrected confidence intervals) further confirms that lactose tolerance in 1500 simultaneously mediates (LLCI = .08, ULCI = .24) and amplifies (LLCI = .12, ULCI = .36) the effect of steady rain on empowering resources in 1800 (*R*^2^ = .78).

**Figure 4.**
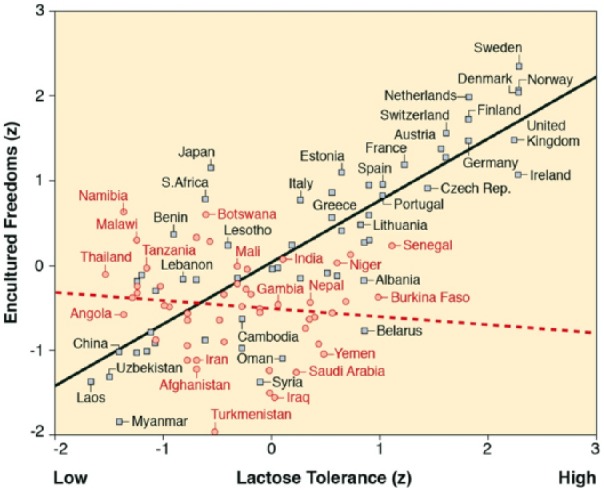
Interactive effects of lactose tolerance in 1500 and empowering resources in 1800 on encultured freedoms in 2000. *Note.* Represented are results while controlling for thermo-hydraulic stress (*R*^2^ = .643). Lactose tolerance has no effect where empowering resources are few (circles and broken horizontal slope for 51 Old World countries; *r* = −.117, *p* = .413), but a strong effect where empowering resources are many (squares and solid upward slope for 57 Old World countries; *r* = .824, *p* < .001; the 51 to 57 split is chosen because other splits provide less telling illustrations of the regression equation)^1^.

Two centuries on, encultured freedoms prevail especially in countries with moderate climatic stress owing to cold/wet weather conditions (Table 4, Model 1). In addition, lactose tolerance in 1500 mediates both the interactive impact of cold and heat and the main effect of steady rain on freedoms today (Table 4, Model 2). Most importantly, empowering resources in 1800 reinforce the positive effect of lactose tolerance in 1500 on encultured freedoms in 2000 (Table 4, Models 3 and 4). Conditional process analysis (Hayes, 2013) is used again, this time to confirm that empowering resources in 1800 simultaneously mediate (LLCI = .04, ULCI = .20) and amplify (LLCI = .07, ULCI = .41) the effect of lactose tolerance in 1500 on encultured freedoms in 2000 (*R*^2^ = .79). As a result of this self-reinforcing process, the prevalence of encultured freedoms today is high especially in countries with *both* greater lactose tolerance *and* greater historic empowering resources (Figure 4).

Because many entwined factors in Table 4 compete and interact with one another to explain encultured freedoms, we also look at the relative importance of the seven predictors—the proportionate contribution each predictor makes to the *R*^2^. Following Johnson and LeBreton’s (2004) recommendation, we consider both each predictor’s direct effect as well as its effect when controlled for the other variables in the regression equation. As can be seen in Table 5, and as perhaps might have been expected, there is a clear split between the combined importance of the three indicators of recent gene-culture coevolution (.67 for empowering resources, lactose tolerance, and their interaction, in this order) and the combined importance of the four indicators of past gene-culture coevolution (.33 for heat stress, steady rain, cold stress, and the interaction of cold stress and heat stress, in this order).

**Table 5.** Relative Importance of Seven Predictors of Encultured Freedoms, Listed in the Order of Decreasing Importance.

Predictor	Relative importance		
	First step^a^	Last step^b^	*M* ^c^
1. Empowering resources in 1800 (ER)	.22	.32	.27
2. Lactose tolerance in 1500 (LT)	.20	.22	.21
3. LT × ER	.20	.18	.19
4. Heat stress (HS)	.10	.14	.12
5. Steady rain	.18	.00	.09
6. Cold stress (CS)	.08	.06	.07
7. CS × HS	.02	.08	.05

a Proportionate contribution if entered separately in the first step of the regression equation.

b Proportionate contribution if entered separately in the last step of the regression equation.

c Mean based on consistent rank orders in the first and in the last step (*r*_s_ = .74, *p* = .06).

The findings are unaffected by weighing countries for geographic and cultural proximity within climato-economic regions (Table 3, Model 5; Table 4, Model 5). The results are also robust against dozens of potentially confounding influences on freedoms, starting with parasitic diseases, which arguably have a substantive influence on human cultural evolution (Acemoglu et al., 2001; Bloom & Sherman, 2005; Fincher & Thornhill, 2012). However, the contemporaneous prevalences of both nonzoonotic and zoonotic diseases only have a negligible impact over and above the impact of the initial predictors (Table 6, Models 1 and 2). Similarly, the reported results are also insensitive to other competing influences, including language fractionalization, ethnic diversity, and religious heterogeneity in the 1980s (Model 3), colonial past (Model 4), state antiquity (Model 5), and current levels of societal industrialization (Model 6), urbanization (Model 7), shadow economy (Model 8), and income inequality (Model 9).

**Table 6.** Effects of Lactose Tolerance in 1500 (LT) and Empowering Resources in 1800 (ER) on Encultured Freedoms in 2000 (Model 1) Compared With Competing Influences (Models 2 to 9).

Regression model	Model 1	Model 2	Model 3	Model 4	Model 5	Model 6	Model 7	Model 8	Model 9
Number of countries	108	108	105	107	99	79	108	81	90
Predictor^a^	*B* ^b^	*B*	*B*	*B*	*B*	*B*	*B*	*B*	*B*
Cold stress (CS)	−.139	−.010	−.096	.015	−.113	−.114	−.164	−.147	−.050
Heat stress (HS)	−.236*	−.216*	−.224*	−.228*	−.158	−.323**	−.244*	−.160	−.131
CS × HS	−.169	−.131	−.100	−.132	−.172	−.050	−.173	−.145	−.163
Steady rain	−.015	−.018	−.091	−.009	.040	−.071	−.017	−.035	−.065
LT	.235***	.287***	.344***	.277***	.250**	.154	.230**	.219*	.289***
ER	.310**	.338***	.317***	.348***	.219*	.275**	.297***	.319***	.335***
LT × ER	.163**	.154*	.141*	.142*	.143*	.175*	.159**	.178**	.168**
Nonzoonotic diseases^c^		.150							
Zoonotic diseases		.047							
Language fractionalization^d^			.209*						
Ethnic diversity			−.139						
Religious heterogeneity			.117*						
Colonial past^e^				.450**					
State antiquity^f^					.015*				
Societal industrialization^g^						.157*			
Urbanization^h^							.002		
Shadow economy^i^								.001	
Income inequality^j^									.018**
Δ*R*^2^	.643***	.015	.046**	.023**	.015*	.019*	.002	.000	.027**
Total *R*^2^	.643***	.658***	.690***	.666***	.687***	.718***	.645***	.703***	.702***

a Predictors are standardized variables and products of standardized variables.

b *B*s shown are unstandardized regression coefficients. There is no multicollinearity (Variance inflation factors ≤ 5.750), and there are no outliers (Cook’s distances ≤ .273).

c The contemporaneous prevalence of nonzoonotic and zoonotic diseases is retrieved from Fincher and Thornhill (2012).

d Language fractionalization, ethnic diversity, and religious heterogeneity in the 1980s are retrieved from Alesina, DeVleeschauwer, Easterly, Kurlat, and Wacziarg (2003).

e Colonial past (no = 0, yes = 1) is retrieved from National Geographic Society (1999).

f According to Schwartz (2009), “The longer a viable state has existed in the territory that currently constitutes a country, the more opportunity there has been for . . . development of secondary institutions in the wider society (e.g., formal governments, schools, courts, hospitals, armies, large corporations)” (pp. 6-7). Adopting that insight, Putterman’s (2004) state antiquity index is used as a proxy for state antiquity.

g Each country’s position on the historical continuum from agriculture to industrial and service employment is proxied by the national percentages of current employment in the agrarian sector (agriculture, fishing, and hunting), the industrial sector (manufacturing, mining, building, and public utilities), and the service sector (trade, transport, restaurants, hotels, finances, communications, and community and personal services) (sources: UNDP, 2004, 2007). The three employment percentages load on a single factor that accounts for 73% of the common variation and represents the extent to which each country is engaged in industrial and service activities.

h The percentage of the total population living in urban areas, as defined by the country (source: Parker, 1997).

i Informal “gray” or “underground” income through concealed economic activities to avoid taxes, social security contributions, obligatory regulations, etc., retrieved from the World Bank (Schneider, Buehn, & Montenegro, 2010).

j The Gini index measures inequality over the entire distribution of income or consumption (source: UNDP, 2004).

* *p* < .05. **p < .01. ***p < .001. (two-sided tests).

## General Discussion

The results of our two-part study suggest a biologically and psychologically motivated process of gene-culture coevolution (Figure 1), which amends the prominent views that economic growth and “good institutions” drive freedoms. However, this pattern of findings is no exception to the rule that every investigation has inherent shortcomings as a result of the methods employed. Thus, it seems appropriate to start with a brief overview of the strengths and weaknesses of the study before discussing main conclusions, implications, and perspectives.

### Strengths and Weaknesses

The strength of simulating the temporality of a specific form of gene-culture coevolution came with the weakness of covering only the last five centuries. The strength of capturing dairying climates in nuances of cold, heat, and rain came with the weakness of having to use 20th-century proxies of spatial climate relations among Old World countries. The strength of concentrating on the mediating and modifying influences of gene-based variation in lactose tolerance on the enculturation of freedoms came with the weakness of neglecting the historical influences of migration (Acemoglu et al., 2001; Acemoglu & Robinson, 2012), warfare (Ross, 1993), and disasters (Oishi & Komiya, 2017). The strength of parsimoniously treating today’s cultural freedoms as an entity came with the weakness of blurring finer evolutionary trajectories of emancipative values, freedom from discrimination, press freedom, and political rights and civil liberties.

To illustrate the last weakness, consider the following study on two kinds of freedom. Conway et al. (2017) made a distinction between vertical restrictions of freedoms by select persons imposing asymmetrical laws on others, and horizontal restrictions through laws that constrain most members of a society equally. They then showed that much ecological stress (heat stress, parasite stress, and frontier topography stress) tends to increase vertical restrictions and decrease horizontal restrictions of freedoms. Cold stress, by contrast, leads to less vertical restrictions of freedoms, especially in rich countries, and to more horizontal restrictions of freedoms irrespective of the country’s level of wealth. So, Conway et al.’s (2017) study about the ecological origins of freedoms exposes the incompleteness of the present exploration by directing full attention to the co-occurring cultural dynamics of freedoms for some and freedoms for all. We strongly encourage more time- and location-sensitive analyses of such processes at different time and geographic scales.

## Main Conclusions

Our discoveries integrate multiple dissociated areas of knowledge. The hot/dry condition that links thermal stress in the *presence* of hydraulic stress to lactose tolerance, seems to be a paralyzing threat that discourages lactose-tolerant people to expand their resources and freedoms. Yet, this finding should not be simplistically read as evidence of climatic determinism, as is exemplified by the cases of Bahrain and Kuwait, where technologies are in place that at least eradicate some stressors. By contrast, the cold/wet condition that links thermal stress in the *absence* of hydraulic stress to lactose tolerance, stands out as a mobilizing challenge that encourages lactose-tolerant people to expand their resources and freedoms. Especially among populations where the role of climate-induced lactose tolerance in 1500 has gradually been integrated into, and complemented with, the leverage-widening role of empowering resources in 1800, are freedoms firmly encultured today (Figure 4; for example, Dutch, Norwegians, Czechs, Swiss).

The results portrayed in Figure 1 indeed suggest the existence of a process of gene-culture coevolution that goes beyond economic and institutional drivers of freedom. Particularly telling in this regard is the fact that some economically successful populations which have come to enjoy greater empowering resources (e.g., Thai, Turks, Emiratis) nevertheless show little tendency to enculture freedoms—to the extent that climatically induced subsistence modes resulting in lactose tolerance did not set the conditions that would more easily translate favorable economic circumstances into emancipative freedoms. Taken together, the findings illustrate the possibility and, indeed, the necessity of building broad interdisciplinary explanations of the complex evolutionary pathways to fundamental forms of human functioning. In the specific case at hand (Table 5), distal climatic and genetic conditions provide a modest partial explanation of why proximate socio-economic resources facilitate greater freedom in some populations but not in others.

### Implications and Perspectives

Perhaps the most important implication of our thermo-hydraulic theory of freedoms is that processes of gene-culture coevolution and niche construction are triggered and shaped, at least in part, by the thermo-hydraulic climate. Ultimately, eternal cold, eternal heat, and perennial aridity cannot create life in the form we know it, leaving no opportunity for culture-mediated dairying, gene-mediated lactose tolerance, empowering resources, and encultured freedoms. In short, it is an axiom, not a falsifiable hypothesis, that climates provide a major context for the emergence of culture. Nevertheless, we have to be careful not to fall into the fallacy of climatic determinism because our ancestors have created trillions of cultural ideas, practices, and artifacts, including property and money, to survive and thrive in cold and hot seasons and places.

Following in the footsteps of the ancient Greeks, who started to dispute the conundrum of climate-induced culture, numerous scholars have focused on the impacts of average temperature and average precipitation on personality, values and practices (for overviews, see Feldman, 1975; Hsiang, Burke, & Miguel, 2013; Parker, 1995; Van Lange, Rinderu, & Bushman, 2017; Wei et al., 2017). Breaking away from this simplistic idea, the empirically supported thermo-hydraulic origins of cultural freedoms in Figure 1 highlight the crucial importance of annual climate fluctuations. Our successful predictions of freedoms seem to confirm that thermal stresses as antecedents of culture are best measured with a new thermometer that indicates downward cold deviations and upward heat deviations from 22°C (pictured in Van de Vliert, 2017) and, likewise, that stressful fluctuations between too little and too much rain influence culture through hydraulic stress.

Spatial variations in culture are pervasive and geographically systematic, yet the origins of any such differences are difficult to pinpoint. In line with a burgeoning literature (e.g., Fischer, 2018; Henrich, 2015; Kong, 2016; Van Lange et al., 2017), we argue that the emergence and diversification of the spatiality of cultural values, beliefs and practices require empirical investigation of the complex interplay between ecological, biological, and psychological processes. Cultural inventions and innovations in specific climatic niches set into motion a millennia-long trajectory that is still shaping our modern living conditions and habits. Political and other societal interventions need to reckon with these climatic conditions that shaped our genes and culture, if they are to be successful in addressing improvements of social life today including promoting freedoms for everyone everywhere.

## Supplemental Material

Supplemenatry_Table – Supplemental material for Got Milk? How Freedoms Evolved From Dairying Climates Click here for additional data file. Supplemental material, Supplemenatry_Table for Got Milk? How Freedoms Evolved From Dairying Climates by Evert Van de Vliert, Christian Welzel, Andrey Shcherbak, Ronald Fischer, and Amy C. Alexander in Journal of Cross-Cultural Psychology

## Notes

1. This split has been freely chosen to do justice to the unique differences between computation and communication in terms of goals, means, and outcomes. Whereas the purpose of the interaction analysis is to estimate the size of the effect (a quantitative goal), the purpose of the interaction plot is to convey the meaning of the effect (a qualitative goal). Whereas the tool of the interaction analysis is the regression equation (a statistical means), the tool of the interaction plot is the visual and verbal clarification of the message (a communicational means). Whereas the interaction analysis leads to an inference (a decisional outcome), the interaction plot leads to an understanding (a learning outcome). This particular split serves a qualitative rather than quantitative goal, uses a communicational rather than statistical means, and entertains a learning rather than decisional outcome.
