# Supplementary material for: Got Milk? How Freedoms Evolved From Dairying Climates
Source: J Cross Cult Psychol. 2018 Jun 11;49(7):1048–65. doi: 10.1177/0022022118778336 (PMC6056908; doi:10.1177/0022022118778336)
Supplement: Supplemenatry_Table – Supplemental material for Got Milk? How Freedoms Evolved From Dairying Climates [file Supplemenatry_Table.pdf]

Supplementary Material for: Got Milk? How Freedoms Evolved from Dairying Climates

Evert Van de Vliert, Christian Welzel, Andrey Shcherbak, Ronald Fischer, and Amy C. Alexander

Table S1. Cold Stress, Heat stress, Steady Rain, and Lactose Tolerance in 1500 for 108 Countries.

Country	Cold Stress [a]	Heat Stress [b]	Minimum Precipitation [c]	Maximum Precipitation [d]	Steady Rain [e]	Lactose Tolerance [f]
Afghanistan	62	18	0	102	0.000	0.25
Albania	44	18	32	211	0.152	0.62
Algeria	33	22	0	137	0.000	0.30
Angola	9	21	0	117	0.000	0.09
Armenia	71	18	8	53	0.151	0.31
Austria	67	16	39	84	0.464	0.79
Azerbaijan	71	18	8	53	0.151	0.31
Bangladesh	15	29	2	437	0.005	0.55
Belarus	91	10	36	88	0.409	0.62
Belgium	64	15	53	95	0.558	0.80

Benin	4	22	13	366	0.036	0.20
Bosnia and Herzegovina	61	19	33	87	0.379	0.57
Botswana	38	29	0	107	0.000	0.27
Bulgaria	63	15	28	87	0.322	0.43
Burkina Faso	16	42	0	277	0.000	0.65
Burundi	7	15	5	125	0.040	0.49
Cambodia	8	31	7	257	0.027	0.35
Cameroon	13	23	23	295	0.078	0.19
China	61	21	3	243	0.012	0.08
Congo-Brazzaville	13	25	0	292	0.000	0.12
Congo-Kinshasa	11	24	3	222	0.014	0.12
Croatia	63	20	46	96	0.479	0.62
Czech Republic	75	16	18	68	0.265	0.76
Denmark	72	11	32	71	0.451	0.96
Egypt	26	34	0	5	0.000	0.27
Estonia	87	11	36	73	0.493	0.57
Ethiopia	37	17	5	300	0.017	0.39
Finland	87	11	36	73	0.493	0.85
France	57	18	35	64	0.547	0.71

Gabon	8	26	3	373	0.008	0.12
Gambia	16	30	0	500	0.000	0.43
Georgia	47	18	15	75	0.200	0.58
Germany	68	16	38	76	0.500	0.85
Ghana	8	26	15	178	0.084	0.25
Greece	35	21	6	71	0.085	0.55
Guinea	6	23	3	1300	0.002	0.39
Guinea-Bissau	10	34	0	254	0.000	0.59
Hungary	67	17	33	72	0.458	0.63
India	23	30	3	180	0.017	0.44
Indonesia	4	26	43	300	0.143	0.36
Iran	54	21	3	46	0.065	0.25
Iraq	34	30	0	28	0.000	0.42
Ireland	61	8	45	74	0.608	0.96
Italy	41	18	15	129	0.116	0.48
Japan	36	16	48	234	0.205	0.28
Jordan	37	23	0	74	0.000	0.23
Kazakhstan	84	20	5	15	0.333	0.23
Kenya	28	13	15	211	0.071	0.17

Kuwait	21	34	0	28	0.000	0.50
Kyrgyzstan	81	16	23	102	0.225	0.18
Laos	19	29	3	302	0.010	0.02
Latvia	87	11	36	73	0.493	0.55
Lebanon	27	23	0	191	0.000	0.22
Lesotho	47	17	8	91	0.088	0.32
Liberia	11	19	31	996	0.031	0.16
Libya	26	30	0	94	0.000	0.41
Lithuania	87	11	36	73	0.493	0.61
Macedonia	61	19	32	61	0.525	0.56
Madagascar	31	18	8	300	0.027	0.34
Malawi	32	18	0	218	0.000	0.12
Malaysia	5	28	99	292	0.339	0.34
Mali	17	39	0	348	0.000	0.34
Moldova	74	13	30	91	0.330	0.42
Mongolia	115	14	0	76	0.000	0.13
Morocco	32	31	0	86	0.000	0.16
Mozambique	20	36	13	130	0.100	0.12
Myanmar	9	31	3	582	0.005	0.08

Namibia	39	17	0	79	0.000	0.09
Nepal	28	16	3	373	0.008	0.50
Netherlands	65	12	40	71	0.563	0.85
Niger	17	40	0	188	0.000	0.56
Nigeria	7	30	25	460	0.054	0.35
Norway	77	12	26	95	0.274	0.96
Oman	11	38	0	28	0.000	0.44
Pakistan	31	28	12	258	0.047	0.51
Philippines	8	28	13	432	0.030	0.36
Poland	77	13	31	96	0.323	0.63
Portugal	33	18	3	111	0.027	0.66
Romania	74	19	26	121	0.215	0.46
Rwanda	18	7	7	183	0.038	0.52
Saudi Arabia	32	31	0	25	0.000	0.47
Senegal	11	34	0	254	0.000	0.68
Serbia	63	20	46	96	0.479	0.48
Slovenia	63	20	46	96	0.479	0.63
Somalia	8	22	0	97	0.000	0.23
South Africa	39	24	8	89	0.090	0.27

South Korea	64	15	20	376	0.053	0.28
Spain	52	17	11	53	0.208	0.66
Sri Lanka	7	23	69	371	0.186	0.25
Sudan	20	44	0	71	0.000	0.41
Sweden	76	13	25	76	0.329	0.96
Switzerland	67	16	64	136	0.471	0.80
Syria	38	23	0	43	0.000	0.39
Tajikistan	66	19	3	66	0.045	0.14
Tanzania	20	23	0	152	0.000	0.14
Thailand	11	34	5	305	0.016	0.05
Tunisia	34	29	3	64	0.047	0.16
Turkey	69	16	10	48	0.208	0.34
Turkmenistan	53	20	5	23	0.217	0.29
Uganda	17	21	46	175	0.263	0.23
Ukraine	74	13	30	91	0.330	0.54
United Arab Emirates	19	34	0	36	0.000	0.37
United Kingdom	55	12	37	64	0.578	0.95
Uzbekistan	53	20	5	23	0.217	0.06
Vietnam	16	32	18	343	0.052	0.35

Yemen	3	30	0	23	0.000	0.53
Zambia	26	22	0	231	0.000	0.11
Zimbabwe	35	17	0	196	0.000	0.15

[a] Cold stress is the sum of the absolute downward deviations in centigrade from 22°C for the average lowest temperature in the coldest month, the average highest temperature in the coldest month, the average lowest temperature in the hottest month, and the average highest temperature in the hottest month (source: Van de Vliert, 2013b; also downloadable from *www.rug.nl/staff/e.van.de.vliert* by clicking on *Projects*). M = 40.472, SD = 26.703.

[b] Heat stress is the sum of the absolute upward deviations in centigrade from 22°C for the average lowest temperature in the coldest month, the average highest temperature in the coldest month, the average lowest temperature in the hottest month, and the average highest temperature in the hottest month (source: Van de Vliert, 2013b). M = 21.889, SD = 7.973.

[c] Minimum monthly precipitation in mm (source: Parker, 1997). M = 16.200, SD = 18.930.

[d] Maximum monthly precipitation in mm (source: Parker, 1997). M = 175.250, SD = 182.733.

[e] Steady rain is the minimal monthly precipitation divided by the maximal monthly precipitation. M = 0.157, SD = 0.190.

[f] Lactose tolerance in 1500 is retrieved from Cook (2014). M = 0.415, SD = 0.239.

Table S2. Empowering Resources in 1800 and Encultured Freedoms in 2000 for 108 Countries.

Country	Empowering Resources in 1800				Encultured Freedoms in 2000				
	Health [a]	Wealth [b]	Postponed Parenthood[c]	Index [d]	1 [e]	2 [f]	3 [g]	4 [h]	Index [i]
Afghanistan	-1.050	-0.712	-1.020	-0.927			-0.715	-1.723	-1.219
Albania	0.912	-0.210	1.678	0.793	-0.512	-0.617	0.269	0.175	-0.171
Algeria	-0.874	0.294	-1.012	-0.531	-1.034	-0.702	-0.476	-0.359	-0.643
Angola	-1.409	-1.276	-0.940	-1.208			-0.371	-0.770	-0.571
Armenia	0.996	-1.172	-1.917	-0.698	-0.599	-0.559	-0.316	0.152	-0.330
Austria	0.658	1.597	1.116	1.123	1.230	0.767	1.482	2.039	1.379
Azerbaijan	0.727	-0.690	-2.255	-0.739	-0.686	-1.381	-0.786	-0.737	-0.898
Bangladesh	-1.875	-0.184	-0.682	-0.914	-0.686	-1.053	-0.520	0.041	-0.554
Belarus	1.112	-0.549	-1.019	-0.152	-0.163	-0.722	-1.342	-0.846	-0.769
Belgium	2.021	1.763	1.397	1.727	0.707	1.370	1.595	1.428	1.275
Benin	-0.249	-0.381	0.610	-0.006		-0.297	0.819	0.585	0.369
Bosnia and Herzegovina	0.836	-0.631	0.206	0.137	-0.163	1.424	0.389	-0.013	0.409
Botswana	0.450	-1.019	-0.422	-0.330		0.995	0.509	0.294	0.600
Bulgaria	1.013	0.476	1.048	0.846	0.185	-1.121	0.481	0.362	-0.023
Burkina Faso	-0.758	-0.792	0.071	-0.493	-0.947		0.313	-0.476	-0.370

Burundi	-0.112	-0.823	-0.794	-0.576			-0.638	-0.826	-0.732
Cambodia	0.811	0.407	-0.966	0.084		-1.073	-0.363	-0.455	-0.630
Cameroon	-0.874	-0.522	0.625	-0.257		-0.297	-0.366	-0.768	-0.477
China	0.025	0.818	0.666	0.503	-0.338	-0.499	-1.693	-1.571	-1.025
Congo-Brazzaville	0.212	-1.123	0.475	-0.145			0.221	-0.591	-0.185
Congo-Kinshasa	0.212	-1.123	0.475	-0.145			-1.074	-0.981	-1.027
Croatia	1.088	1.272	0.386	0.915	0.794	-0.633	0.447	0.510	0.280
Czech Republic	0.811	1.853	0.861	1.175	0.794	0.103	1.351	1.433	0.921
Denmark	0.811	1.460	2.311	1.527	2.013	2.414	1.751	2.045	2.056
Egypt	0.293	0.246	0.037	0.192	-1.121	-0.831	-0.858	-0.695	-0.876
Estonia	1.186	0.307	0.568	0.687	0.620	1.394	1.569	0.802	1.096
Ethiopia	-0.615	-0.149	-1.210	-0.658	0.533	-1.073	-1.272	-0.357	-0.542
Finland	-0.030	0.924	1.318	0.737	1.578	1.508	1.792	2.048	1.731
France	0.132	1.529	1.891	1.184	1.143	1.319	1.266	1.040	1.192
Gabon	-0.361	-1.177	-0.457	-0.665			-0.343	-0.292	-0.318
Gambia	-0.874	-0.854	-0.470	-0.733		0.220	-1.073	-0.524	-0.459
Georgia	0.458	-0.945	-1.917	-0.801	-0.686	-0.891	0.118	-0.230	-0.422
Germany	1.646	1.944	0.779	1.456	1.752	1.550	1.577	0.988	1.467
Ghana	-1.108	-0.572	-0.378	-0.686	-1.034	0.866	1.047	0.461	0.335

Greece	1.211	0.706	0.071	0.663	1.143	-0.099	1.162	1.250	0.864
Guinea	-0.672	-1.185	-0.019	-0.625			-0.384	-0.643	-0.514
Guinea-Bissau	0.025	-1.364	-1.304	-0.881			0.201	0.051	0.126
Hungary	1.063	1.532	1.026	1.207	0.098	-0.108	1.186	1.186	0.590
India	-1.907	-0.347	0.161	-0.698	-0.686	-0.290	0.609	0.711	0.086
Indonesia	-0.529	-0.535	0.026	-0.346	-0.686	-0.628	-0.100	0.317	-0.274
Iran	-1.844	0.249	-1.109	-0.901	-0.686	-1.287	-1.667	-0.818	-1.115
Iraq	-0.194	0.106	-1.168	-0.419	-1.557		-1.077	-2.004	-1.546
Ireland	1.479	1.249	2.127	1.618	0.533	0.701	1.438	1.621	1.073
Italy	-0.815	1.455	0.700	0.447	0.794	0.122	0.754	1.425	0.774
Japan	1.162	0.959	2.261	1.461	1.056	1.224	1.131	1.193	1.151
Jordan	-0.057	-0.477	-0.984	-0.506	-1.557	-0.955	-0.546	-0.712	-0.942
Kazakhstan	-1.655	0.021	-0.401	-0.678	-0.425	-0.648	-0.778	-0.658	-0.627
Kenya	-1.875	-0.540	-0.915	-1.110		-0.039	-0.368	-0.301	-0.236
Kuwait	-1.717	-0.010	-0.871	-0.866	-0.773	-0.820	0.016	-0.929	-0.626
Kyrgyzstan	0.754	-1.164	-0.547	-0.319	-0.250	-0.426	-0.527	-0.642	-0.461
Laos	-0.003	0.245	-0.041	0.067			-1.349	-1.378	-1.363
Latvia	0.293	-0.456	1.003	0.280	0.185	0.159	1.197	0.698	0.560
Lebanon	-0.615	0.701	0.397	0.161	-0.076		-0.168	-0.244	-0.163

Lesotho	0.239	-1.252	0.284	-0.243			0.290	0.195	0.243
Liberia	-0.221	-0.415	-0.109	-0.248			-0.176	-0.421	-0.299
Libya	0.293	-0.321	-1.241	-0.423	-1.295		-1.675	-1.534	-1.502
Lithuania	-0.844	0.986	0.372	0.171	0.272	-0.404	1.171	0.862	0.475
Macedonia	0.458	0.386	0.217	0.354	0.011	-0.936	0.362	0.073	-0.123
Madagascar	-0.389	-0.886	-0.895	-0.723		-0.814	0.022	0.138	-0.218
Malawi	-0.445	-1.275	-0.772	-0.831		0.608	-0.015	0.326	0.306
Malaysia	-0.361	0.250	0.723	0.204	-0.163	0.412	-0.546	-0.257	-0.139
Mali	-1.594		-0.154	-0.874		-1.202	0.812	0.382	-0.002
Moldova	0.458	-0.734	-0.333	-0.203	-0.250	0.118	-0.213	0.229	-0.029
Mongolia	-0.030	-0.190	0.172	-0.016		-1.202	0.324	0.541	-0.112
Morocco	0.319	-0.604	-0.457	-0.247	-1.034	-1.596	-0.453	-0.536	-0.905
Mozambique	-0.445	-1.287	-0.603	-0.778		-0.943	0.180	0.060	-0.234
Myanmar	-0.305	-0.324	0.071	-0.186			-1.950	-1.733	-1.841
Namibia	0.132	-1.334	0.150	-0.351		0.758	0.817	0.337	0.637
Nepal	0.239	-0.959	-0.064	-0.261			-1.015	0.154	-0.430
Netherlands	1.998	2.677	1.105	1.926	1.665	2.448	1.777	2.059	1.987
Niger	-0.305	-1.211	-0.828	-0.781			0.089	-0.019	0.035
Nigeria	-0.417	-0.298	-0.513	-0.409	-1.295	-0.196	-0.333	-0.107	-0.483

Norway	1.741	0.741	1.996	1.492	2.362	2.100	1.780	2.061	2.076
Oman	0.105	0.588	-0.878	-0.062			-0.833	-1.352	-1.093
Pakistan	-1.748	0.002	-0.558	-0.768	-1.470	0.499	-0.680	-0.777	-0.607
Philippines	-0.277	-0.123	-0.401	-0.267	-0.076	-0.716	0.105	0.522	-0.041
Poland	1.038	1.223	-0.210	0.684	0.446	-0.525	0.702	0.597	0.305
Portugal	0.962	1.367	1.790	1.373	0.098	0.138	1.600	1.467	0.826
Romania	0.988	1.034	0.981	1.001	-0.076	-0.100	0.376	0.764	0.241
Rwanda	-0.030	-1.224	-1.103	-0.786	-0.773		-1.028	-0.961	-0.921
Saudi Arabia	0.052	-0.852	-0.861	-0.554	-0.686		-1.285	-1.789	-1.253
Senegal	-1.970	-0.951	0.442	-0.827			0.376	0.099	0.238
Serbia	0.937		0.217	0.577	0.185	-1.077	0.448		-0.148
Slovenia	1.211	1.483	1.307	1.334	1.665	-0.134	1.095	1.188	0.954
Somalia	-0.700	-0.598	-0.878	-0.725			-1.253	-0.973	-1.113
South Africa	0.424	0.274	-0.423	0.092	0.446	0.759	1.092	0.833	0.782
South Korea	-1.780	-0.228	0.105	-0.634	0.272	-0.298	0.763	0.414	0.288
Spain	-0.672	1.609	1.082	0.673	0.969	0.523	1.076	1.274	0.960
Sri Lanka	0.185	-0.795	0.464	-0.049		-0.039	-0.521	0.049	-0.170
Sudan	-0.139	-0.657	-0.614	-0.470			-1.083	-1.382	-1.232
Sweden	0.788	1.267	2.269	1.441	2.710	2.907	1.728	2.060	2.351

Switzerland	1.551	1.840	2.194	1.862	1.665	1.526	1.702	1.363	1.564
Syria	-0.221	0.510	-0.853	-0.188			-1.262	-1.472	-1.367
Tajikistan	0.781	-0.631	0.251	0.133		-1.460	-0.831	-0.748	-1.013
Tanzania	0.078	-0.719	-0.727	-0.456	-0.425	0.382	0.105	-0.171	-0.027
Thailand	-0.417	-0.605	-0.288	-0.437	-0.338	-0.320	-0.217	0.501	-0.094
Tunisia	-0.874	-0.516	-0.345	-0.578	-1.121	-0.556	-1.141	-0.736	-0.888
Turkey	0.811	0.558	-0.929	0.147	-0.512	-0.226	-0.057	0.185	-0.152
Turkmenistan	-1.666	-0.101	-0.142	-0.636			-2.024	-1.890	-1.957
Uganda	-1.939	-0.761	-0.895	-1.198	-0.947	-0.729	-0.113	-0.444	-0.558
Ukraine	1.211	-0.161	-1.580	-0.177	-0.250	-0.168	-0.340	0.418	-0.085
United Arab Emirates	-0.333	0.499	-0.951	-0.262		0.737	-0.472	-1.272	-0.336
United Kingdom	1.882	2.924	1.262	2.022	1.491	1.783	1.361	1.275	1.478
Uzbekistan	0.781	-1.334	0.431	-0.041	-0.947		-1.512	-1.473	-1.311
Vietnam	0.025		1.565	0.795	-0.512	-0.451	-1.334	-1.570	-0.966
Yemen	-2.559		-0.884	-1.722	-1.644		-1.030	-0.454	-1.042
Zambia	0.185		-0.693	-0.254	-0.076	-0.860	-0.379	-0.165	-0.370
Zimbabwe	0.476		-0.738	-0.131	-0.860	0.168	-1.905	-0.505	-0.775

[a] Health is the standardized inverse of child mortality in 1800, retrieved on February 2, 2015, from https://www.gapminder.org. N = 108.

[b] Wealth is the standardized income per capita in 1800, retrieved on February 2, 2015, from https://www.gapminder.org. $N = 102$.

[c] Postponed parenthood is the standardized inverse fertility in 1800, retrieved on February 2, 2015, from https://www.gapminder.org. $N = 108$.

[d] Index is the average of health in 1800, wealth in 1800, and postponed parenthood in 1800. $N = 108$, $M = -0.005$, $SD = 0.814$. The three measures load on one single factor (Eigenvalue $\lambda = 1.98$, $R^2 = 0.661$; internal consistency in terms of Cronbach's $\alpha = 0.742$).

[e] Standardized contemporaneous emancipative values emphasizing free choice and equal opportunities, retrieved from Welzel (2013). $N = 73$.

[f] Standardized contemporaneous freedom from discrimination is the inverse positive discrimination of relatives and fellow nationals, retrieved from Van de Vliert (2011a). $N = 80$.

[g] Standardized contemporaneous press freedom is the inverse repression of journalists and media assistants, retrieved from Van de Vliert (2011b). $N = 108$.

[h] Standardized political rights and liberties are the Unified Democracy Scores, retrieved from Pemstein, Meserve and Melton (2010). $N = 107$.

[i] Index is the average of the measures 1 to 4. $N = 108$, $M = -0.091$, $SD = 0.900$. These measures capture a strong overall index of reciprocal and mutually reinforcing freedoms ($0.640 < r < 0.907$, p's < 0.001), and load on one single factor (Eigenvalue $\lambda = 3.208$, $R^2 = 0.802$; internal consistency in terms of Cronbach's $\alpha = 0.930$).
